# Hes1 promotes cell proliferation and migration by activating Bmi-1 and PTEN/Akt/GSK3β pathway in human colon cancer

**DOI:** 10.18632/oncotarget.5484

**Published:** 2015-10-06

**Authors:** Fei Gao, Wei Huang, YuQin Zhang, ShaoHui Tang, Lin Zheng, Feng Ma, YiMing Wang, Hui Tang, Xin Li

**Affiliations:** ^1^ Department of Gastroenterology, The First Affiliated Hospital of Jinan University, Guangzhou 510630, China; ^2^ Cancer Research Institute, Southern Medical University, Guangzhou 510515, China; ^3^ Department of Oncology, The First Affiliated Hospital of Jinan University, Guangzhou 510515, China; ^4^ Clinical Experimental Center, The First Affiliated Hospital of Jinan University, Guangzhou 510630, China; ^5^ Department of Pathology, School of Basic Medical Sciences, Southern Medical University, Guangzhou 510515, China

**Keywords:** Hes1, Bmi-1, PTEN, metastasis, colon cancer

## Abstract

Hes1 is a transcription factor that influences cell proliferation and differentiation. However, the effect of Hes1 on invasiveness and the underlying mechanism remain unknown. In the current study, we found that Hes1 suppressed cell apoptosis, promoted cell growth, induced EMT phenotype and cytoskeleton reconstruction, and enhanced the metastatic potential of colon cancer cells *in vitro* and *in vivo*. Furthermore, we indicated that Bmi-1 mediated Hes1-induced cell proliferation and migration, downregulated PTEN and activated the Akt/GSK3β pathway, consequently induced EMT and cytoskeleton reconstruction, ultimately leading to enhanced invasiveness of cancer cells. In addition, we also found that both Hes1 and Bmi-1 could directly regulate PTEN by associating at the PTEN locus, and played important roles in regulating PTEN/Akt/GSK3β pathway. Our results provide functional and mechanistic links between Hes1 and Bmi-1/PTEN/Akt/GSK3β signaling in the development and progression of colon cancer.

## INTRODUCTION

Colon cancer is one of the most frequent cancers in the world. Studies have demonstrated an increase in the incidence of colon cancer and this trend will continue, at least because of increasing life expectancy [1]. Although the majority of patients can undergo initially curative local resection, leading causes of death are local recurrence and metastasis [2].

Epithelial-mesenchymal transition or transformation (EMT), a traditional phenomenon revealed in embryonic development, has been gradually accepted as a potential mechanism underlying cancer progression and metastasis. Evidences suggest that EMT plays critical roles in different aspects of cancer progression, such as metastasis, stem cell features, and chemoresistance [3].

Hes1 is a transcription factor that influences cell proliferation and differentiation in embryogenesis. Studies have indicated that Hes1 influences the maintenance of certain stem cells and progenitor cells and partially influences the digestive systems through the Notch signalling pathway, i.e., Hes-Notch interactions play a role in digestive organ development [4]. In addition, Hes1 acts as a marker of normal colon stem cells [5], however, an increase in Notch signalling, including the Notch target Hes1, may contribute to the initiation of colon cancer [6]. Our previous study showed that hes1 was involved in the self-renewal and tumourigenicity of stem-like cancer cells in colon cancer [7]. Since the roles of Hes1 in the pathogenesis of colon cancer are not well elucidated, these aforementioned findings prompted us to investigate whether Hes1 is related to the cell growth, apoptosis and migration of human colon cancer and the potential mechanisms.

Bmi-1 (B cell-specific MLV inte-gration site-1) is an important member of polycomb group genes (PcG). It has been reported that Bmi-1 plays an important role in the stem cell proliferation, differentiation and aging in several types of cancer, such as bladder, skin, prostate, breast, ovarian, colorectal as well as hematological malignancies, and is related to a series of pathological processes in these cancers, such as invasion, metastasis and prognosis [8]. It has also been reported that Bmi-1 represses the tumor suppressor PTEN and induces EMT in some cancers [9].

Herein, we demonstrate that overexpression of Hes1 suppresses cell apoptosis, promotes cell growth, migration in colon cancer cells. While knockdown of Hes1 enhances cell apoptosis, reduces cell growth and cell invasion. Most importantly, we show that Bmi-1 is a direct target of Hes1. Hes1 binds to the Bmi-1 locus and upregulates Bmi-1expression, which consequently downregulates PTEN and activates the Akt/GSK3β pathway, induces EMT and cytoskeleton reconstruction, ultimately leads to enhanced invasiveness of cancer cells. Besides, Hes1 could also directly regulate PTEN expression by associating at the PTEN locus. Taken together, our results indicate that Hes1 plays a quantitative role in the development and progression of colon cancer and suggest a mechanism that links Hes1 to Bmi-1/PTEN/Akt/GSK3β pathway.

## RESULTS

### Hes1 promotes cell growth in colon cancer

**Figure 1:**
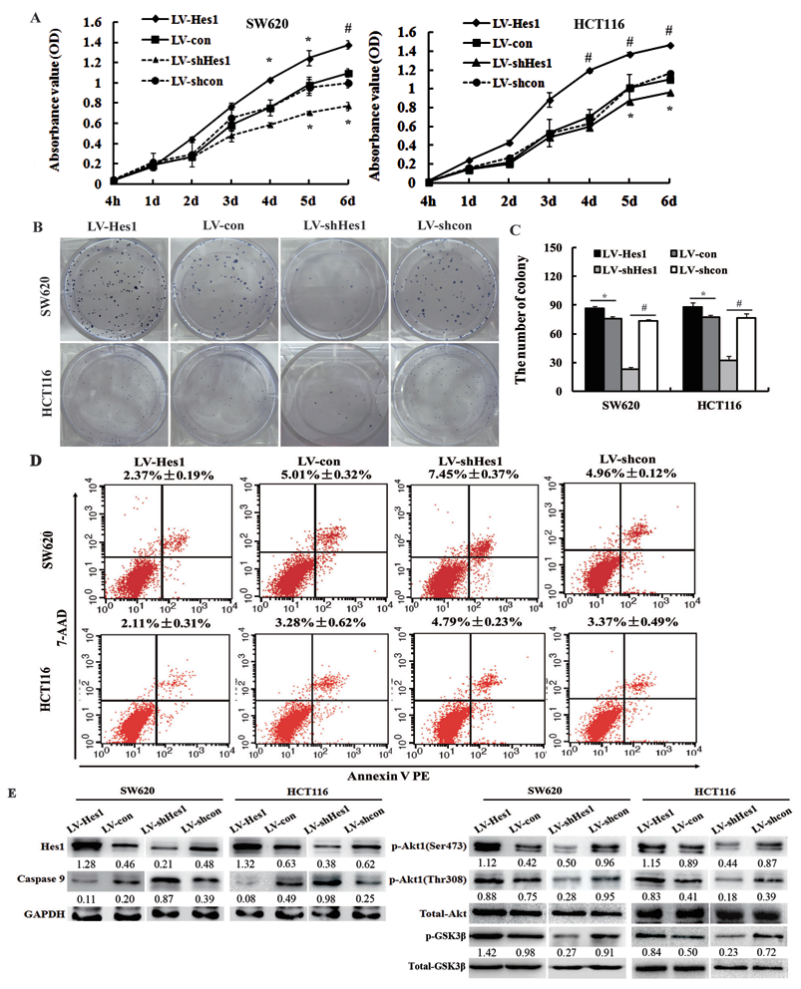
Effect of Hes1 on the cell growth and apoptosis of colon cancer cells **A.** Effects of Hes1 over-expression or inhibition on colon cancer cell growth by MTT assay. **B, C.** Effects of Hes1 over-expression or inhibition on colon cancer cell growth by colony formation assay. **D.** Detection of apoptosis by the Annexin V PE/7-AAD using a flowcytometer. **E.** The effects of Hes1 on the expression of p-Akt1, p-GSK3β and caspase 9 determined by western blotting. *P < 0.05; ^#^*P* < 0.01, as compared LV-Hes1 with LV-con groups, or LV-shHes1 with LV-shcon groups.

As shown in Figure S2, results of Microarray demonstrated an increased expression of Hes1 in colon cancer biopsy samples compared with control normal samples, which was confirmed by qPCR shown in the latter part of the article. To explore the effect of Hes1 on cell growth, SW620 and HCT116 cells were stably transfected with Hes1 (LV-Hes1) or Hes1 shRNA (LV-shHes1), respectively. As shown in Figure 1A, results of MTT assay displayed that Hes1 promoted cell growth in SW620 and HCT116 cells, whereas Hes1 shRNA inhibited cell growth in colon cancer cells (*P* < 0.05 or 0.01). By contrast, Hes1 control (LV-con) or Hes1 shRNA control (LV-shcon) had no effect on cell growth, indicating that the effect caused by Hes1 was highly specific.

Figure 1B and Figure 1C showed the results of colony formation assay, LV-Hes1–infected SW620 and HCT116 cells displayed more and bigger colonies compared with LV-con–infected cells, whereas LV-shHes1-infected cells displayed much fewer and smaller colonies compared with LV-shcon-infected cells.

### Effects of Hes1 on colon cancer cell apoptosis and Akt activation

As shown in Figure 1D, Annexin V-PE Apoptosis Detection Kit showed that Hes1 overexpression decreased the rate of apoptosis in SW620 and HCT116 cells to 2.37% ± 0.19% and 2.11% ± 0.31%, respectively, compare to the control cells, 5.01% ± 0.32% and 3.28% ± 0.62%, respectively. By contrast, Hes1 inhibition increased the rate of apoptosis in SW620 and HCT116 cells to 7.45% ± 0.37% and 4.79% ± 0.23%, respectively, compare to the control cells 4.96% ± 0.12% and 3.37% ± 0.49%, respectively. Results of the caspase 9 activity by western blotting were also shown in Figure 1E. Caspase 9 was upregulated in Hes1 inhibited colon cancer cells, whereas down-regulated in Hes1 over-expressed cells.

We next analyzed Hes1-mediated effects on the Akt/GSK3β pathway because of its known anti-apoptotic and EMT-promoting role [11], Akt1 is well known to be activated by phosphorylation at Ser473 and Thr308. We therefore examined the Akt activation status in colon cancer cells by western blotting using phosphorylated Akt1 (p-Akt1) antibodies, which recognizes only phosphorylated Akt1 at Ser473 and Thr308. We evaluated the expressed amount of total Akt protein, p-Akt1 protein and Hes1 protein in Hes1 over-expressed and inhibited SW620 and HCT116 cells (Figure 1E). The expressed amount of total Akt protein in Hes1 over-expressed or inhibited cells and the corresponding control cells was almost the same level. In contrast, p-Akt1 expression in Hes1-expressed colon cancer cells was observed to increase significantly in comparison with that in the control cells. However, a decreased p-Akt1 expression was observed in Hes1-inhibited colon cancer cells in comparison with that in the control cells. What's more, since the activation of Akt leads to the phosphorylation of GSK3β, which is active in resting cells, but is inactivated by the phosphorylation, we found activated Akt and phosphorylation of GSK3β herein (Figure 1E).

### Hes1 promotes EMT and enhances the invasiveness of colon cancer cells, while silencing Hes1 represses the EMT phenotype and reduces transformation and metastatic potential of colon cancer cells

**Figure 2:**
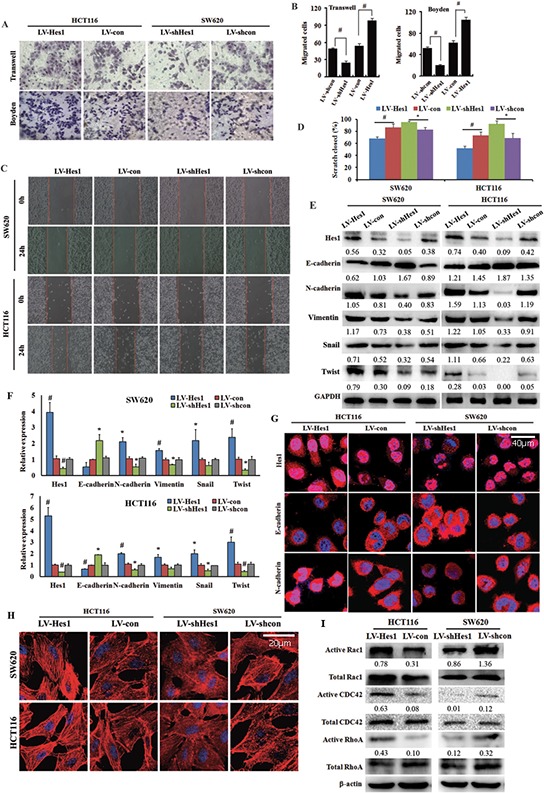
Effect of Hes1 expression on colon cancer cell migration and cell cytoskeleton organization **A, B.** Transwell and Boyden assay. The invasive cells were stained and counted under microscope at 24–30 h after reseeding. Original magnification, × 400. ^#^*P* < 0.01, as compared LV-Hes1 with LV-con groups, or LV-shHes1 with LV-shcon groups. **C, D.** Wound healing assay. Representative images photographed at 0 h (upper) and 24 h (lower) post-wounding. Original magnification, × 200. **E.** EMT-related genes detected by western blotting. **F.** EMT-related genes detected by qPCR. **G.** E-cadherin and N-cadherin showed by immunofluorescence. **H.** Cytoskeleton measured by phalloidin staining. **I.** The expression of Rac1, CDC42 and RhoA detected by western blotting.

It was reported that expression of Hes1 was associated with invasive and metastatic in osteosarcoma cells [12]. Thus, we investigated effects of Hes1 on cell invasion and motility in colon cancer cells by conducting assays for Transwell chamber and Matrigel-coated Boyden chamber invasion and wound healing. As shown in Figure 2A and 2B, Hes1-expressing SW620 and HCT116 cells exhibited significantly increased mobility compared with control cells, while Hes1-silencing SW620 and HCT116 cells decreased mobility (*P* < 0.01). The result was confirmed by scratch migration assay (Figure 2C and 2D).

In order to determine whether Hes1 induces EMT, we probed the cells with epithelial marker E-cadherin and mesenchymal marker N-cadherin and Vimentin, as well as Snail and Twist, two well-known EMT-related genes. As shown in Figure 2E and 2F, Hes1 exhibited a typical EMT phenotype, including downregulation of E-cadherin and upregulation of N-cadherin, Vimentin, Snail and Twist. The EMT phenotype was confirmed by immunofluorescent staining (Figure 2G).

It has been illustrated that cytoskeleton is associated with EMT and cytoskeletal reorganization is a prerequisite for cell motility and cancer cell invasion [13, 14, 15]. We observed that, the stress fibre formation (stained with phalloidin stain) was increased in Hes1-expressing cells, but decreased in shHes1-expressing cells (Figure 2H).

Since it is well established that the actin cytoskeleton is regulated by Rho GTPases and the three prototypic Rho GTPases, RhoA, Rac1 and Cdc42, are best known for their effects on the actin cytoskeleton [16]. Herein, pull-down analyses showed up-regulated active Rac1, CDC42 and RhoA expression in Hes1-expressing HCT116 cells, whereas down-regulated active Rac1, CDC42 and RhoA expression in Hes1-silencing SW620 cells (Figure 2I).

To confirm the results, we showed that the knockdown of overexpressed Hes1 by siRNA reduced the acute effects, while transfection of RNAi-resistant Hes1 constructs recover the effects observed in shHes1-expressing cells, such as cell migration (Figure S3A, S3B), cadherin expression (Figure S3C), cytoskeletal organization (Figure S3D) and Rho GTPase activation (Figure S3E).

Taken together, our results suggest that upregulation of Hes1 is sufficient to induce EMT and enhance invasiveness of colon cancer cells *in vitro*, moreover, Hes1 induces cytoskeleton reconstruction of colon cancer cells.

### Hes1 expression is associated with Bmi-1 expression in colon cancer

**Figure 3:**
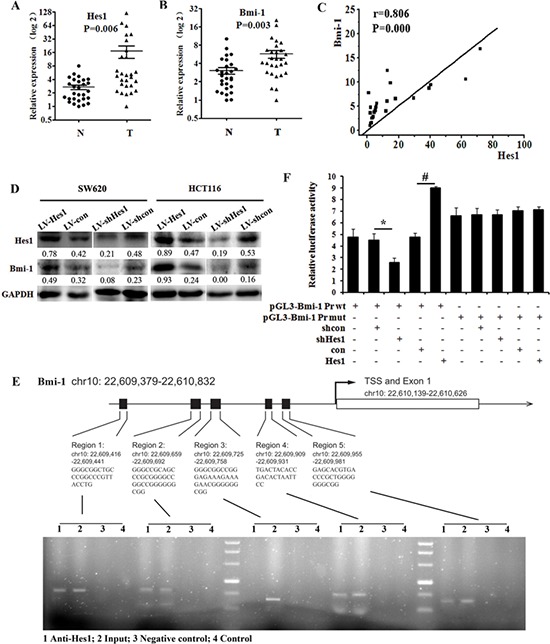
Hes1 expression is associated with Bmi-1 expression in colon cancer **A.** The expression of Hes1 in colon cancer biopsy samples and control normal samples detected by qPCR. **B.** The expression of Bmi-1 in colon cancer biopsy samples and control normal samples detected by qPCR. *N* = normal, *T* = tumor. **C.** Hes1 correlates positively with Bmi-1 in colon cancer tissue samples. **D.** Effect of Hes1 on the expression of Bmi-1. **E.** Binding of Hes1 to the Bmi-1 promoter detected by CHIP. **F.** Luciferase reporter assays in HCT116 cells, with cotransfection of wt or mt Bmi-1 locus including region 4 and 5 as indicated. *P < 0.05, #*P* < 0.01 compared with control.

We predicted Hes1 could affect the promoter sequence of Bmi-1 by bioinformatic methods (data not shown). To determine whether any correlation exists between Hes1 expression and the expression of Bmi-1 in colon cancer biopsy samples, we obtained RNA from 28 normal samples and 28 colon cancer samples and analysed the expression levels of Hes1 and Bmi-1 through qRT-PCR. Results showed that Hes1 and Bmi-1 were upregulated in the colon cancer tissue compared with the normal samples (Figure 3A, 3B). We also found that Hes1 expression was positively correlated with the expression of Bmi-1 (Figure 3C). We subsequently found up-regulated Bmi-1 expression in Hes1-expressing SW620 and HCT116 cells, and down-regulated Bmi-1 expression in Hes1-silencing cells (Figure 3D).

To investigate direct binding between Hes1 and Bmi-1, we carried out a CHIP assay, results indicated that Hes1 increased the expression of Bmi-1 by associating at the Bmi-1 locus (Figure 3E). Results were confirmed by the Luciferase reporter assays, shown in Figure 3F.

### PTEN is directly regulated by both Bmi-1 and Hes1 in colon cancer

**Figure 4:**
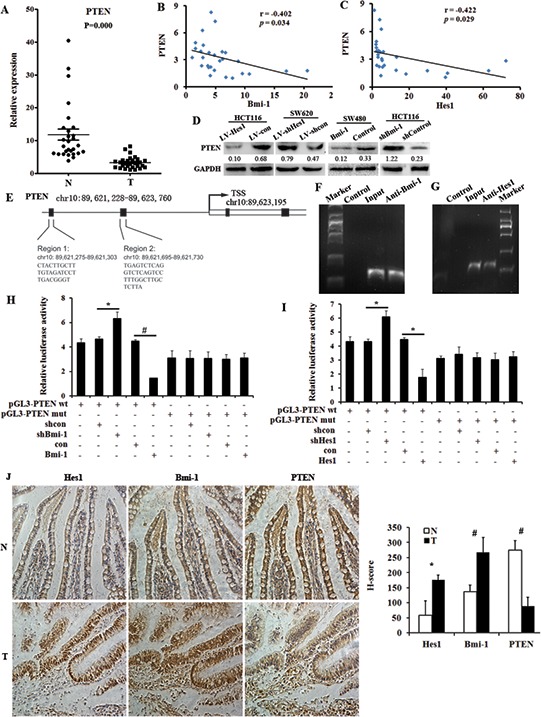
PTEN is directly regulated by Bmi-1 and Hes1 in colon cancer **A.** The expression of Hes1 in colon cancer biopsy samples and control normal samples detected by qPCR. **B.** PTEN correlates negatively with Bmi-1 in colon cancer tissue samples. **C.** PTEN correlates negatively with Hes1 in colon cancer tissue samples. **D.** The expression of PTEN in Hes1 or Bmi-1 overexpressing and silencing colon cancer cells. **E.** The promoter and possible locus of PTEN. **F.** Binding of Bmi-1 to the PTEN promoter (region 2) detected by CHIP. **G.** Binding of Hes1 to the PTEN promoter (region 2) detected by CHIP. **H, I.** Luciferase reporter assays in HCT116 cells, with cotransfection of wt or mt PTEN locus including region 1 and 2 as indicated. **J.** The expression of Hes1, Bmi-1 and PTEN in colon cancer biopsy samples and control normal samples showed by immunohistochmeistry and serial section. Original magnification, × 400. *P < 0.05, #*P* < 0.01 compared with control.

Since it has been reported that Bmi-1 represses the tumor suppressor PTEN by associating at the PTEN locus [9], and Hes1 could effect on the promoter sequence of PTEN [17]. We then detected the expression of PTEN in colon cancer biopsy samples and control normal samples, as shown in Figure 4A, we found downregulated PTEN expression in colon cancer samples. In addition, PTEN expression was found to be negatively correlated with the expression of Bmi-1 and Hes1 in colon cancer tissue samples, Figure 4B and 4C. As shown in Figure 4D and Figure S1A, we also found that PTEN was downregulated in Hes1 or Bmi-1 overexpressing cells and upregulated in Hes1 or Bmi-1 inhibiting cells.

We then analysed whether PTEN was directly regulated by Bmi-1 and Hes1, and found both Bmi-1 and Hes1 could associate at the PTEN locus detected by CHIP (Figure 4E, 4F, 4G). Results were confirmed by Luciferase reporter assays (Figure 4H, 4I). Figure 4J shows the results of the immunohistochemical analysis of sections from human normal colon tissue, Hes1 and Bmi-1 were upregulated in cancer samples compared to their expression in normal samples, while PTEN was downregulated in cancer samples. In addition, PTEN reduced the invasive phenotype and cytoskeleton reorganization of Hes1-expressing colon cancer cells (Figure S4A, S4B, S4C) and restored the Hes1-induced EMT phenomenon in cancer cells (Figure S4D). PTEN also reduced the invasive phenotype and cytoskeleton reorganization of Bmi-1-expressing colon cancer cells (Figure S4E, S4F, S4G) and restored the Bmi-1-induced EMT phenomenon in cancer cells (Figure S4H).

As a result, we conclude that Hes1 and Bmi-1 can directly regulate PTEN by binding the PTEN promoter, and play important roles in regulating PTEN//Akt/GSK3β pathway.

### Hes1 enhances tumorigenicity and metastasis *in vivo*

**Figure 5:**
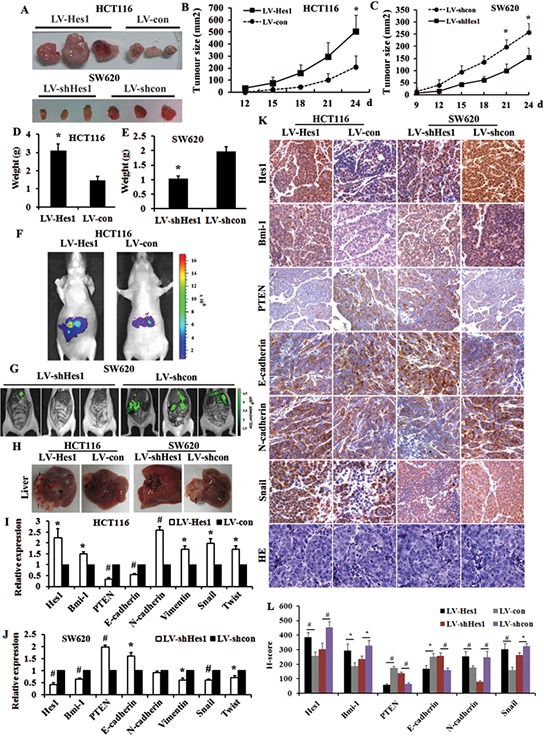
Hes1 enhances tumorigenicity and metastasis of colon cancer cells *in vivo* **A.** Tumors formed after injection of nude mice with LV-Hes1 expressing HCT-116 cells and LV-shHes1 expressing SW620 cells. **B.** Tumor growth curves after injection of nude mice with LV-Hes1 expressing HCT-116 cells. **C.** Tumor growth curves after injection of nude mice with LV-shHes1 expressing SW620 cells. **D.** Tumor weight after injection of nude mice with LV-Hes1 expressing HCT-116 cells. **E.** Tumor weight after injection of nude mice with LV-shHes1 expressing SW620 cells. **F.** Representative images of luciferase signals of mice in each group 4 weeks after intrasplenic injection with LV-Hes1 expressing HCT-116 cells compared with LV-con. **G.** Representative images of luciferase signals of mice Mice after injection with LV-shHes1 expressing SW680 cells compared with LV-shcon detected. **H.** Liver metastasis in intrasplenic injection model, Arrows indicate metastasis nodules. **I.** Expression of EMT-related genes in LV-Hes1 expressing HCT-116 cells detected by qPCR. **J.** Expression of EMT-related genes in LV-shHes1 expressing SW620 cells detected by qPCR. (I) Expression of EMT-related genes in Hes1 overexpressing and silencing colon cancer cells indicated by immunohistochmeistry.

Our previous studies have suggested that Hes1 increases the number of tumor initiating cells *in vivo* [7], in this study, we subsequently observed that injection of HCT116 with a higher Hes1 expression (LV-Hes1) subcutaneously led to larger tumors compared with injection of HCT116 with LV-con cells, but injection of SW620 with LV-shHes1 led to smaller tumors compared with injection of SW620 with LV-shcon cells (Figure 5A, 5B, 5C, 5D, 5E).

To track the behavior of Hes1 in metastasis, we carried out intrasplenic injection model for liver colonization assays. We labeled the LV-Hes1-expressing HCT116 cells and LV-con-expressing HCT116 cells with firefly luciferase and inoculated cells intrasplenically into 5 nude mice separately. As shown in Figure 5F, overexpression of Hes1 resulted in greater liver metastases burden. In contrast, downregulation of endogenous Hes1 in SW620 resulted in inhibition of distant organs metastasis (3 pairs of nude mice). The metastases were all shown as numerous green nodules in LV-shHes and LV-shcon expressing cells (3 pairs of nude mice, Figure 5G). Furthermore, we surveyed the expression levels of EMT-related genes of the metastatic livers through qPCR and immunohistochemistry (Figure 5H, 5I, 5J and 5K), and found higher expression of Hes1, Bmi-1, N-cadherin and Snail, while decreased expression of PTEN and E-cadherin in Hes1-overexpressing groups, moreover, the genes expressed reversely in Hes1-silencing groups. All these results indicated the metastatic ability was significantly increased by Hes1 expression and suppressed by silencing Hes1 expression.

### Bmi-1 is required for Hes1-mediated cell growth promotion and apoptosis inhibition

**Figure 6:**
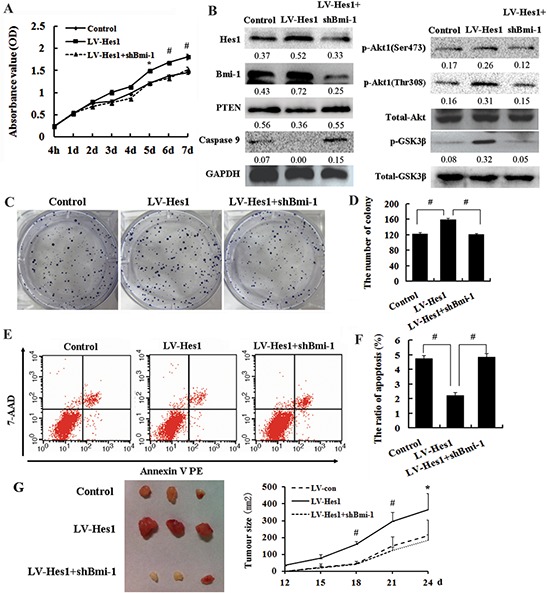
Bmi-1 is required for Hes1-mediated cell growth promotion and apoptosis inhibition **A.** Inhibition of Bmi-1 reduced the accelerated cell growth showed by cell growth curve. **B.** Bmi-1 increased PTEN expression and reduced Akt1 and pGSK3β activation induced by Hes1. **C, D.** Inhibition of Bmi-1 reduced the accelerated cell growth investigated by colony formation assay. **E, F.** Bmi-1 abrogated Hes1 protection of colon cancer cells from apoptosis. **G.** Left panel: Tumors formed after injection of nude mice with LV-Hes1 expressing HCT-116 cells and LV-shHes1+shBmi-1 expressing cells; Right panel: Tumor growth curves.

Since Bmi-1 was confirmed to promote cell proliferation and inhibit cell apoptosis and was involved in the increase of Akt phosphorylation in the human breast carcinoma cell line MCF-7 [18]. We then silenced Bmi-1 expression in Hes1-expressing SW620 cells using RNAi, and found inhibition of Bmi-1 abrogated Hes1 protection of colon cancer cells from apoptosis (Figure 6E, 6F) and reduced the accelerated cell growth *in vitro* and *in vivo* (Figure 6A, 6C, 6D, 6G), we also found Akt activation and GSK3β phosphorylation (Figure 6B) induced by Hes1. In addition, we overexpressed Bmi-1 in SW480 cells and inhibited Bmi-1 in HCT116 cells, and found Bmi-1 could promote cell growth (Figure S1C, S1D, S1E) and activate Akt/GSK3β phosphorylation (Figure S1B).

Together, these results demonstrate that Bmi-1 is important in mediating the cell growth promotion, Akt activation and antiapoptotic function of Hes1 in colon cancer cells.

### Bmi-1 mediates Hes1-induced cell invasion and cytoskeleton reconstruction

**Figure 7:**
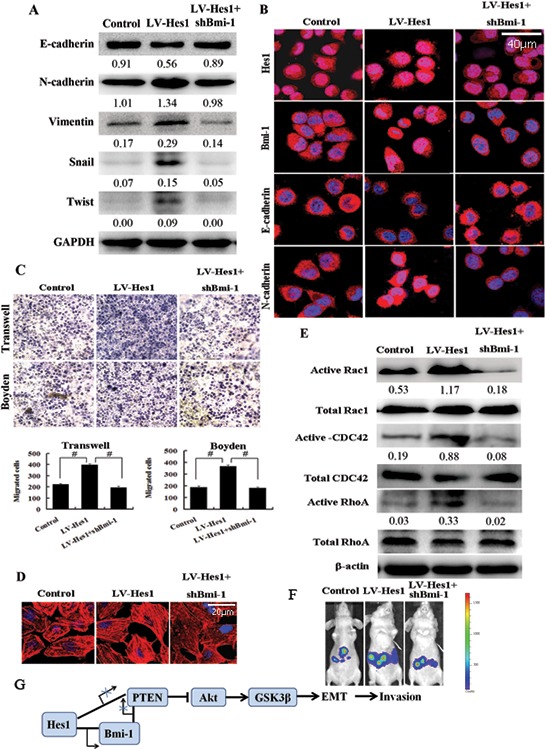
Bmi-1 mediates Hes1-induced cell invasion and cytoskeleton reconstruction **A.** Ablation of Bmi-1 expression restored the Hes1-induced EMT phenomenon detected by Western blotting. **B.** Ablation of Bmi-1 expression restored the Hes1-induced EMT phenomenon showed by immunofluorescence. **C.** The migratory/invasive phenotype using Transwell and Boyden assays by inhibiting Bmi-1 in Hes1-expressing colon cancer cells. Original magnification, × 200. #*P* < 0.01 compared with control. **D.** Bmi-1 mediated Hes1-induced cytoskeleton reconstruction measured by phalloidin staining. **E.** Bmi-1 mediated Hes1-induced changes in the expression of Rac1, CDC42 and RhoA. **F.** Ablation of Bmi-1 expression restored the Hes1-induced metastasis *in vivo*. **G.** A schematic model of Hes1 functions during the invasion-metastasis cascade.

Studies indicated that Bmi-1 promoted the invasion and tumorigenesis and induced EMT in several cancer cells [8, 9, 19]. We then detected the EMT markers expression, the ability of migration and invasion and the cytoskeleton in Hes1-expressing SW620 cells with Bmi-1 inhibited. Western blotting and immunofluorescence showed that ablation of Bmi-1 expression restored the Hes1-induced EMT phenomenon in cancer cells (Figure 7A, 7B). Transwell and Boyden assays indicated that inhibition of Bmi-1 reduced the migratory/invasive phenotype of Hes1-expressing colon cancer cells (Figure 7C), which was confirmed by *in vivo* experiments (Figure 7F). What's more, Bmi-1-silencing could reverse the increased F-actin, up-regulated active Rac1, CDC42 and RhoA expression in Hes1-expressing SW620 cells (Figure 7D, 7E). Besides, as shown in Figure S1A, S1F and S1G, Bmi-1 induced cell invasion and affected EMT-related genes expression.

Taken together, as shown in Figure 7G, Bmi-1 mediates Hes1-induced EMT, cell invasion and cytoskeleton reorganization by activating PTEN/Akt/GSK3β pathway, Hes1 also downregulates PTEN by binding the PTEN locus, all of which consequently induces EMT and cytoskeleton reconstruction, and ultimately leads to enhanced invasiveness of cancer cells.

## DISCUSSION

It has been reported that Hes1 plays an important role in the tumourigenesis of biliary neuroendocrine tumors and blocking of Hes1 expression initiates the differentiation of human neural stem cells and telencephalic progenitor cells [20, 21, 22]. Another study showed that Hes1 might modulate the therapeutic resistance in breast cancer [23]. Herein, we found that Hes1 promoted cell growth in SW620 and HCT116 cells, whereas Hes1-silencing inhibited cell growth in colon cancer cells. Moreover, Hes1 enhanced tumorigenicity and cell growth *in vivo*.

Metastasis is a basic biological characteristic of malignant tumor, and the primary factors affecting patient survival, which has become a hot and difficult field of cancer research. The most prominent feature of tumor metastasis is the tissue specificity, which is different tumor can form metastases in the same or different parts of the human body [24]. Epithelial-mesenchymal transition or transformation (EMT) is a process characterized by loss of cell adhesion, repression of E-cadherin expression, and increased cell motility [25]. EMT is an important contributor to the invasion and metastasis of epithelial-derived cancers. Many patients present with metastatic (stage IV) colorectal cancer at the time of diagnosis. It was reported that expression of Hes1 was associated with invasive and metastatic potential in osteosarcoma [12]. In this study, we found that Hes1 promoted EMT and enhanced the invasiveness of colon cancer cells, while silencing Hes1 repressed the EMT phenotype and reduced transformation and metastatic potential of colon cancer cells *in vitro* and *in vivo*. As mentioned above, cytoskeleton is associated with EMT and cytoskeletal reorganization is a prerequisite for cell motility and cancer cell invasion, and members of Rho GTPases including RhoA, Rac1 and Cdc42 play an important role in EMT as well [15]. Specifically, RhoA induces formation of actin stress fibers and cell-cell adhesions, whereas Rac1 stimulates formation of lamellipodia [26]. This study demonstrated up-regulated active Rac1, CDC42 and RhoA expression in Hes1-expressing colon cancer cells, whereas down-regulated active Rac1, CDC42 and RhoA expression in Hes1-silencing cells, which indicated that Hes1 induced cytoskeleton reconstruction of colon cancer cells. Thus, we conclude that Hes1 might play an important role in the tumourigenesis, metastasis and development of colon cancer.

EMT endows cells with migratory and invasive properties, induces stem cell properties, and prevents apoptosis and senescence [27]. The Akt/GSK3β pathway is one important signal transduction pathway for chemoprevention and cancer treatment studies. Evidence supporting the importance of the Akt in cancer cell growth and apoptosis has been well documented in previous studies [28]. PTEN, a renowned tumor suppressor, functions as a negative regulator of the Akt pathway, and has crucial roles in cell proliferation, survival, differentiation and migration [9, 29]. Here, results indicated that Hes1 downregulated PTEN, and promoted Akt activation and GSK3β phosphorylation in colon cancer cells.

Previous reports have showed that Bmi-1 promotes drug resistance and cellular invasion in cancer cells and cancer stem cells [30, 31], moreover, Hes1 is a well known key apoptosis-inhibiting genes [32, 33]. In this study, we found that Bmi-1 was up-regulated in colon cancer tissues and expressed positively correlated with the expression of Hes1. We also found up-regulated Bmi-1 expression in Hes1-expressing SW620 and HCT116 cells, and down-regulated Bmi-1 expression in Hes1-silencing cells, Hes1 increased the expression of Bmi-1 by associating at the Bmi-1 locus as well. By silencing Bmi-1 with RNAi in Hes1-expressing cells, we found that Bmi-1-silencing abrogated Hes1 protection of colon cancer cells from apoptosis, reduced the accelerated cell growth and Akt activation induced by Hes1. Furthermore, Bmi-1-silencing could reverse EMT, migratory/invasive phenotype and the abnormal actin polymerization caused by Hes1. In addition, Bmi-1 also downregulated PTEN by associating the PTEN locus and consequently activated the Akt/GSK3β pathway, ultimately induced EMT and cytoskeleton reconstruction, leading to enhanced invasiveness of cancer cells. These results suggested a role of Bmi-1 in the cell growth promotion, PTEN/Akt/GSK3βactivation, antiapoptotic function, EMT, cell invasion and cytoskeleton reorganization of Hes1 in colon cancer cells.

Hes1 plays a role in the initiation of colon cancer and is involved in the self-renewal and tumourigenicity of stem-like cancer cells in colon cancer as we have reported previously [7]. However, the complex regulatory machineries of Hes1 during cancer stem cell induction and metastasis promotion have not been fully elucidated. Hence, the elucidation of the underlying signalling network that regulates these Hes1-associated changes of biological behavior, for example, enhanced metastatic potential and cancer stem cell features, will provide important insights into the exact role of Hes1 in colon cancer.

## MATERIALS AND METHODS

### Cell lines and cell culture

Human colon cancer cell lines (SW620, SW480 and HCT116) were cultured in RPMI1640 medium supplemented with 10% foetal bovine serum (FBS) in a humidified incubator with 5% CO_2_ at 37°C.

### Establishment of stable cell lines

The lentivirus vector for Hes1 without GFP was bought from Addgene and the lentivirus vector for Hes1 knockdown with GFP and the control vector were kindly gifts from Dr. Ryoichiro Kageyama. Vectors were transfected into 293FT cells by packaging plasmid PMD2G and PPAX2. Colon cancer cells were stably transfected with Hes1 lentiviruses and were selected with neomycin, and immunofluorescence was carried out to confirm the transfection efficiency (Figure S5A). Colon cancer cells were stably transfected with Hes1 knockdown vector and the clones were selected by GFP expression using flow cytometry, cells were assessed by fluorescence and phase microscopy (Figure S5B). For *in vivo* experiments, cells were stably transfected with pLVX-TRE3G-Luc vecter. Cells were planted on 96-well plates at an initial concentration of 50 cells/well, and allowed to grow overnight with regular growth medium. Then the regular medium was replaced with medium containing the D-luciferin (150 μg/ml). After treated for 2–5 min, bioluminescence images were taken using IVIS Lumina Series III Imaging System (Life Sciences, Hopkinton, MA) (Figure S5C).

The stably overexpressing cell lines were identified using real-time qPCR and western blot.

### Ethics statement

All animal work was conducted under the institutional guidelines of Guangdong Province and approved by the Use Committee for Animal Care. Approval from the Jinan University Institute Research Ethics Committee was obtained, and written informed consent was provided by each human subject.

### Clinical samples

The biopsies of 77 colon cancer patients and 38 non-tumoral tissues were collected from the Department of Pathology at Nanfang Hospital of Guangzhou City in China between 2010 and 2012. None of the patients received preoperative radiotherapy or chemotherapy.

### MTT assay

Cells (1 × 10^3^) were plated onto 96-well plates in 80% growth medium and allowed to adhere overnight. At different time points (4 h, 1d, 2d, 3d, 4d, 5d and 6d), the culture medium was removed and replaced with culture medium containing MTT dye (5 mg/ml). After incubation at 37°C for 4 h, the MTT solution was removed, and dimethyl sulfoxide (DMSO) was added to dissolve the formazan crystals. Spectrometric absorbance at 570 nm was measured by microplate photometer (Thermo, MA).

### Colony formation assay

Cells were were plated in 6-well plates at 200 per well and grown for 2 weeks. After 2 weeks, the cells were washed twice with PBS, fixed with methanol/acetic acid (3:1, v/v), and stained with hematoxylin. Then, the number of colonies was counted.

### Annexin V-PE Apoptosis Detection Kit

Cells were spun down to remove supernatant and resuspended in 100 μl Annexin V binding buffer. Then 5 μl of Annexin V-PE and 5 μl 7-AAD from the Annexin V-PE Apoptosis Detection Kit (Keygen Biotech) were added into the solution according to the guidelines. After 15 min of incubation, 400 μl of Annexin V binding buffer was added. The Annexin V-PE and 7-AAD stained cells were analyzed by the FL2 and FL4 channels.

### RNA isolation, reverse transcription, and qRT-PCR

For the mRNA analyses, the total RNA was extracted using the Trizol Reagent (Invitroen) according to the protocol provided by the manufacturer. The total RNA was reversely transcribed using the PrimeScript RT reagent Kit (TaKaRa). The qRT-PCR for the analysis of mRNA expression was performed on a Stratagene Mx3005P qRT-PCR system using the SYBR Green qRT-PCR master mix (TaKaRa) and GAPDH for normalisation. The primers used for the amplification of the indicated genes are listed in Table S1. All of the samples were normalised to the internal controls, and the fold changes were calculated through relative quantification (2^−ΔΔCt^).

### Western blot analysis

Protein lysates were separated by 10% SDS-PAGE, and electrophoretically transferred to PVDF (polyvinylidene difluoride) membrane (Millipore). The membrane was incubated with antibodies, Hes1 (Bioss), Bmi-1 (Abcam), PTEN (Abcam), Snail (Abcam), Twist (Abcam), Vimentin (Bioss), Akt1 (Santa Cruz), pAkt1-Ser473 (Santa Cruz), pAkt1-Thr308 (Santa Cruz), GSK3β(Santa Cruz), p-GSK3β(Santa Cruz), Caspase 9 (Santa Cruz), E-cadherin (Santa Cruz), N-cadherin (Santa Cruz). The relative amount of active Rac1, CDC42 and RhoA was determined by GTP bound (active) form divided by the amount of total Rac1, CDC42 and RhoA in the whole cell lysate (according to the protocol of RhoA/Rac1/Cdc42 Activation Assay Combo Biochem Kit provided by Cytoskeleton, Inc.). Then the membrane was incubated with HRP-labelled goat-anti-mouse or rabbit IgG, and the proteins were detected by high sensitivity chemiluminescence imaging system (BIORAD). Glyceraldehyde-3-phosphate dehydrogenase (GAPDH) was used as the protein-loading control.

### Immunohistochemistry

The immunohistochemistry analysis was performed as described below. After deparaffinisation and rehydration, the TMA sections were subjected to high pressure for 2 min to achieve antigenic retrieval. The slides were incubated overnight at 4°C with the following primary antibodies: Hes1 (1:200 dilution; Bioss), Bmi-1 (1:500 dilution; Abcam), PTEN (1:500 dilution; Abcam), Snail (1:500 dilution; Abcam), E-cadherin (1:300 dilution; Santa Cruz), N-cadherin (1:300 dilution; Santa Cruz). The sections were then incubated with DAB for 2 min. In every run, the primary antibodies were substituted with PBS for the negative controls. H-score was used to evaluate the IHC results [10].

### Microarray analysis

The total RNA was extracted from normal colon tissues and tumor tissues. Gene expression were determined using Phalanx human OneArray microarrays (HOA 6.1) following the manufacturer's instructions.

### Immunofluorescence analysis

Cell lines were plated on culture slides (Costar, MA) and after 24 hours were rinsed with PBS and fixed in 4% paraformaldehyde for 10 min, then permeabilized with 0.05% triton X-100. The cells were then blocked for 30 min in 10% BSA (Sigma, MO) in PBS and then incubated with primary monoclonal antibodies in PBS for 1 hours at 37°C, antibodies: Hes1 (Bioss), E-cadherin (Santa Cruz), N-cadherin (Santa Cruz). After three washes in PBS, the slides were incubated for 1 h in the dark with secondary goat anti-mouse, or goat anti-rabbit antibodies (Invitrogen, Carlsbad, CA). After three further washes, the slides were stained with 4-,6-diamidino-2-phenylindole (DAPI; Sigma, St. Louis, MO) for 5 min to visualize the nuclei, and examined using a confocal laser-scanning microscope (Olympus FV1000).

For F-actin cytoskeleton, fixed cells were incubated with 200 nM working stock of Acti-stain™ 670 phalloidin (Cytoskeleton). Cells were counterstained with DAPI (Sigma) and imaged with a confocal laser-scanning microscope (Olympus FV1000).

### Cell migration and invasion assays

For the cell migration assay, 1 × 10^5^ cells in 100 μl RPMI 1640 medium without NBCS were seeded on a fibronectincoated polycarbonate membrane insert in a transwell apparatus (Corning, MA). In the lower chamber, 500 μl RPMI 1640 with 10% NBCS was added as chemoattractant. After the cells were incubated for 20–24 h at 37°C in a 5% CO_2_ atmosphere, the insert was washed with PBS, and cells on the top surface of the insert were removed with a cotton swab. Cells adhering to the lower surface were fixed with methanol, stained with Hematoxylin and counted under a microscope in five predetermined fields (200 ×). All assays were independently repeated at least thrice.

For the cell invasion assay, the procedure was similar to the cell migration assay, except that the transwell membranes were precoated with 24 μg/μl Matrigel (BD, USA) and the cells were incubated for 20–24 h at 37°C in a 5% CO2 atmosphere. Cells adhering to the lower surface were counted the same way as for the cell migration assay.

### Scratch migration assay

Cells were scratched using the tip of a sterile 10 μl pipette (width: ~1 mm) in each well. The plates were washed twice with PBS in order to remove the detached cells, and incubated at 37°C in 5% CO2. Wound closure was monitored at various time points by observation under a microscope and the degree of cell migration was quantified by the ratio of gap distance at 24 h to that at 0 h. The experiment was done in triplicate.

### Animal studies

The animal studies were approved by the Institutional Animal Care and Use Committee of Jinan University, Guangzhou, China. Male athymic BALB/c nude mice (4–5 weeks old) were used for animal studies. Subcutaneous tumor growth assays were performed as previously described (Gao et al., 2014). Intrasplenic injection model was used for liver colonization assays. The mice were kept in pathogen-free conditions. The metastases were monitored using the IVIS Lumina Series III Imaging System (Life Sciences, Hopkinton, MA) 5–10 min after intraperitoneal injection of 3 mg of luciferin (Sigma).

### Chromatin immunoprecipitation (ChIP) assay

Aaccording to the protocol of CHIP Assay Kit (Merk Millipore). HCT116 Cells were fixed in 1% formaldehyde at 37°C for 10 min. Cells were washed twice with ice-cold phosphate-buffered saline containing protease inhibitors (1 mM phenylmethylsulfonyl fluoride, 1 g/ml aprotinin, and 1 g/ml pepstatin A), scraped, and pelleted by centrifugation at 4°C. Cells were resuspended in lysis buffer, incubated for 10 min on ice, and sonicated to shear DNA. After sonication, the lysate was centrifuged at 13,000 rpm for 10 min at 4°C. The supernatant was diluted in ChIP dilution buffer (0.01% SDS, 1% Triton X-100, 2 mM EDTA, 16.7 mM Tris-HCl, pH 8.1, 167 mM NaCl, and protease inhibitors). Antibodies were added to the supernatant and incubated overnight at 4°C with rotation. The immunocomplex was collected on protein A/G-agarose and washed sequentially with low salt wash buffer (0.1% SDS, 1% Triton X-100, 2 mM EDTA, 200 Mm Tris-HCl, pH 8.1, and 150 mM NaCl), high salt wash buffer (0.1% SDS, 1% Triton X-100, 2 mM EDTA, 200 mM Tris-HCl, pH 8.1, and 500 mM NaCl), LiCl wash buffer (0.25 M LiCl, 1% Nonidet P-40, 1% deoxycholate, 1 mM EDTA, and 10 mM Tris-HCl, pH 8.1), and finally Tris/EDTA buffer (10 mM Tris-HCl and 1 mM EDTA, pH 8.0). The immunocomplex was eluted with elution buffer (1% SDS, 0.1 M NaHCO3, and 200 mM NaCl), and the cross-links were reversed by heating at 65°C for 4 h. After the reaction, the samples were adjusted to 10 mM EDTA, 20 mM Tris-HCl, pH 6.5, and 40 g/ml proteinase K and incubated at 45°C for 1 h. DNA was recovered and subjected to PCR amplification using the primers specific for the detection of regions containing the sites of the Bmi-1 promoter or PTEN promoter.

## SUPPLEMENTARY FIGURES AND TABLE


