# Supplementary material for: Hes1 promotes cell proliferation and migration by activating Bmi-1 and PTEN/Akt/GSK3β pathway in human colon cancer
Source: Oncotarget. 2015 Oct 6;6(36):38667–80. doi: 10.18632/oncotarget.5484 (PMC4770728; doi:10.18632/oncotarget.5484)
Supplement: Supplementary file 1 [file oncotarget-06-38667-s001.pdf]

SUPPLEMENTARY FIGURES AND TABLE

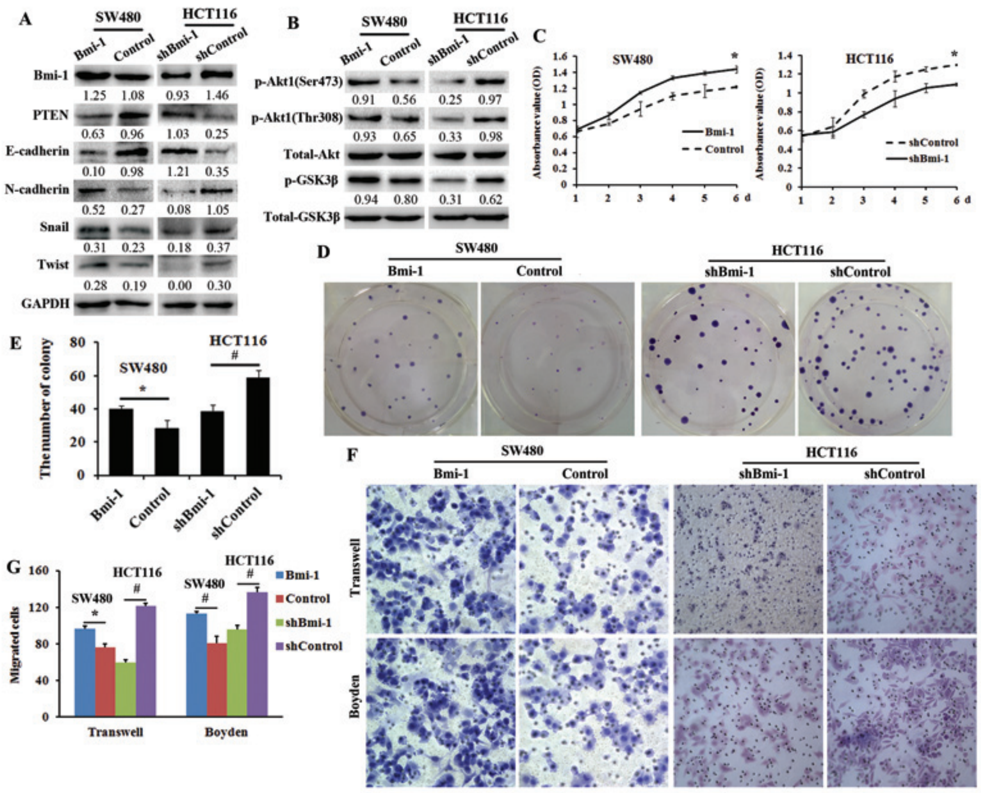

Supplementary Figure S1: Effect of Bmi-1 on cell proliferation and migration. A. Effect of Bmi-1 on the expression of EMT-related genes in colon cancer cells by western blot. **B.** Effect of Bmi-1 on Akt and GSK3β activation in colon cancer cells showed by western blot. **C.** Effect of Bmi-1 over-expression or inhibition on colon cancer cell growth by MTT assay. **D, E.** Effect of Bmi-1 on colon cancer cell growth by colony formation assay. **F, G.** Effect of Bmi-1 on cell migration detected by Transwell and Boyden assay. Original magnification, × 200. *P < 0.05, #P < 0.01 compared with control.

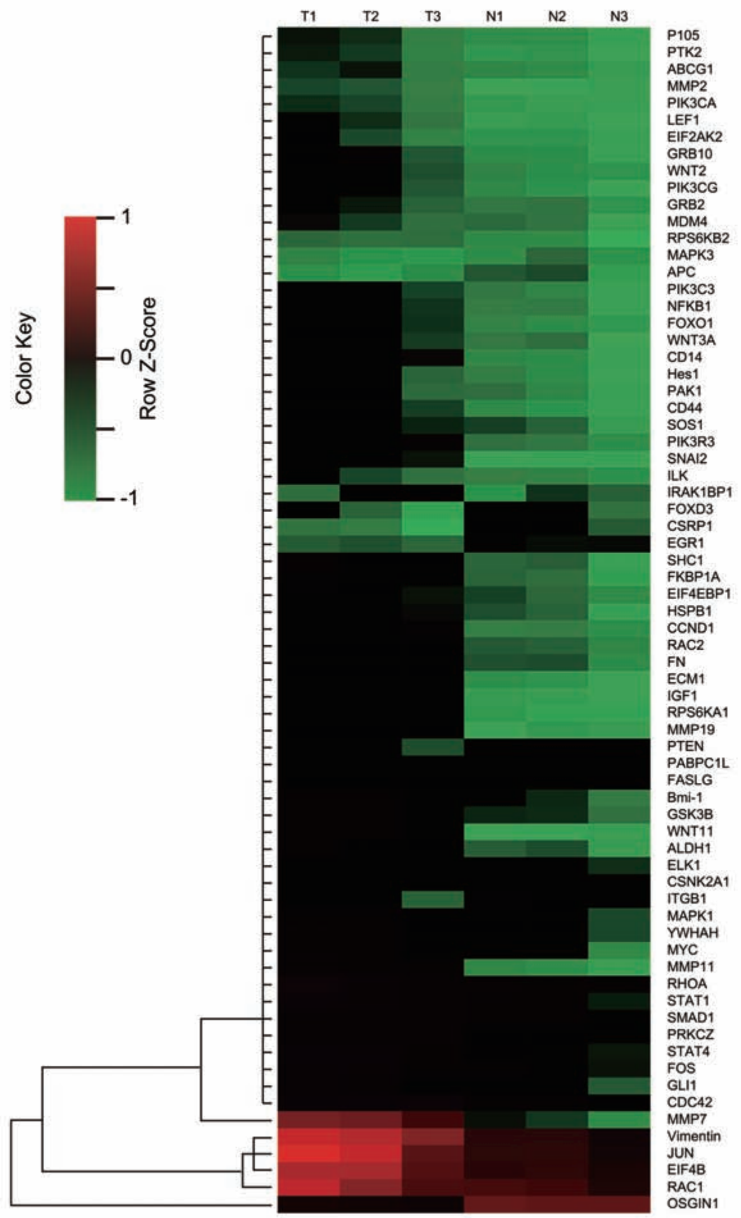

Supplementary Figure S2: Expression of genes in colon cancer biopsy samples and control normal samples detected by Microarray.

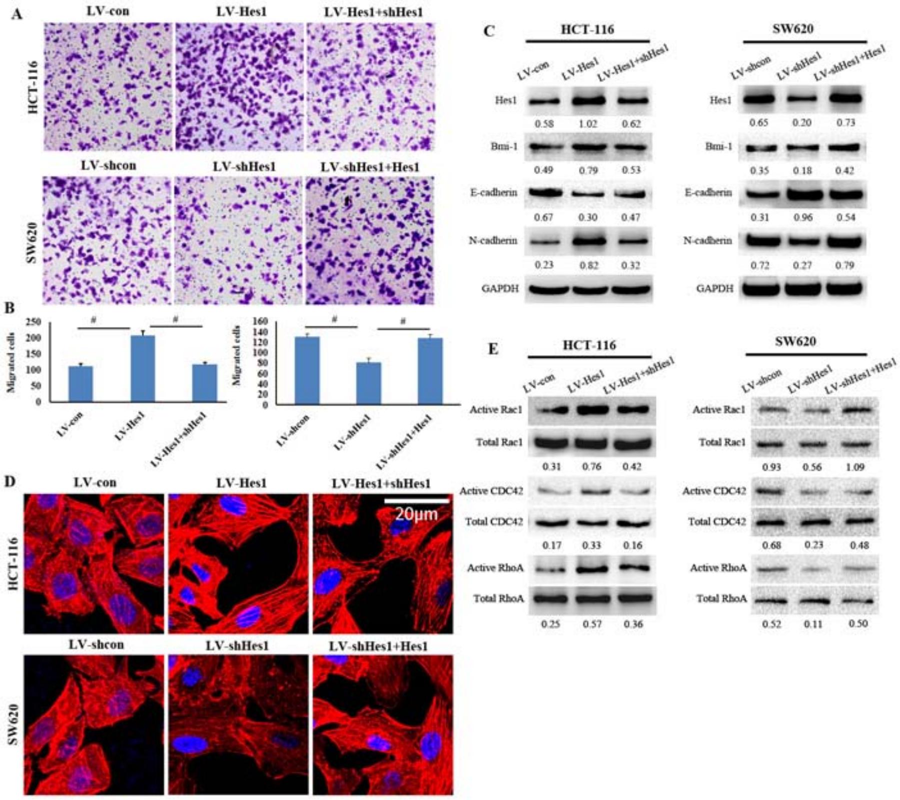

Supplementary Figure S3: Knockdown of overexpressed Hes1 by siRNA reduced the effects induced by Hes1, while transfection of RNAi-resistant Hes1 constructs recover the defects observed in shHes1-expressing cells. A, B. Cell migration. Original magnification, × 200. *$P < 0.05$, $^{\#}P < 0.01$ compared with control. **C.** Cadherin expression. **D.** Cytoskeletal organization. **E.** Rho GTPase activation.

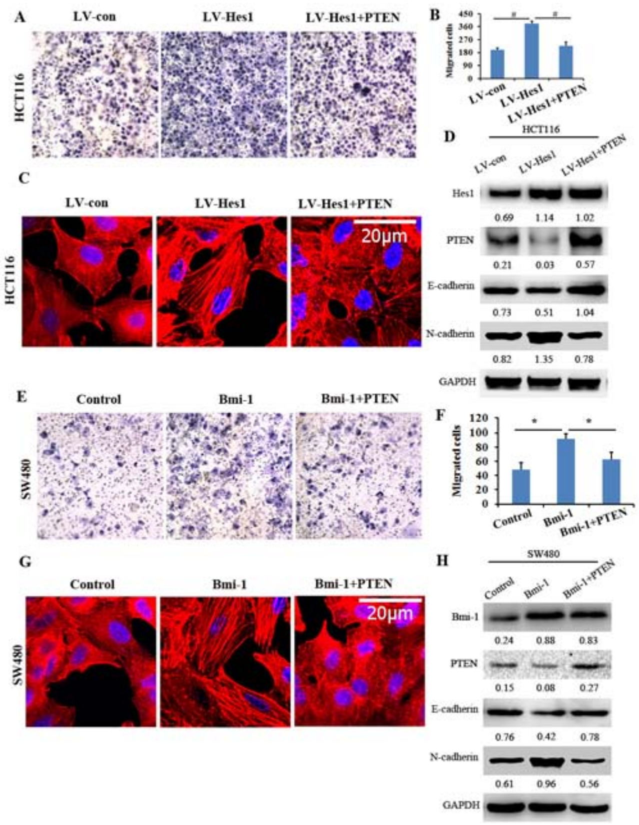

Supplementary Figure S4: PTEN mediated Hes1 and Bmi-1 induced EMT, cell invasion and cytoskeleton reorganization. A, B. PTEN mediated Hes1-induced cell invasion. Original magnification, × 200. **P* < 0.05, #*P* < 0.01 compared with control. **C.** PTEN mediated Hes1-induced cytoskeleton reorganization. **D.** PTEN mediated Hes1-induced EMT using Western blot. **E, F.** PTEN mediated Bmi-1-induced cell invasion. Original magnification, × 200. **P* < 0.05, #*P* < 0.01 compared with control. **G.** PTEN mediated Bmi-1-induced cytoskeleton reorganization. **H.** PTEN mediated Bmi-1-induced EMT using Western blot.

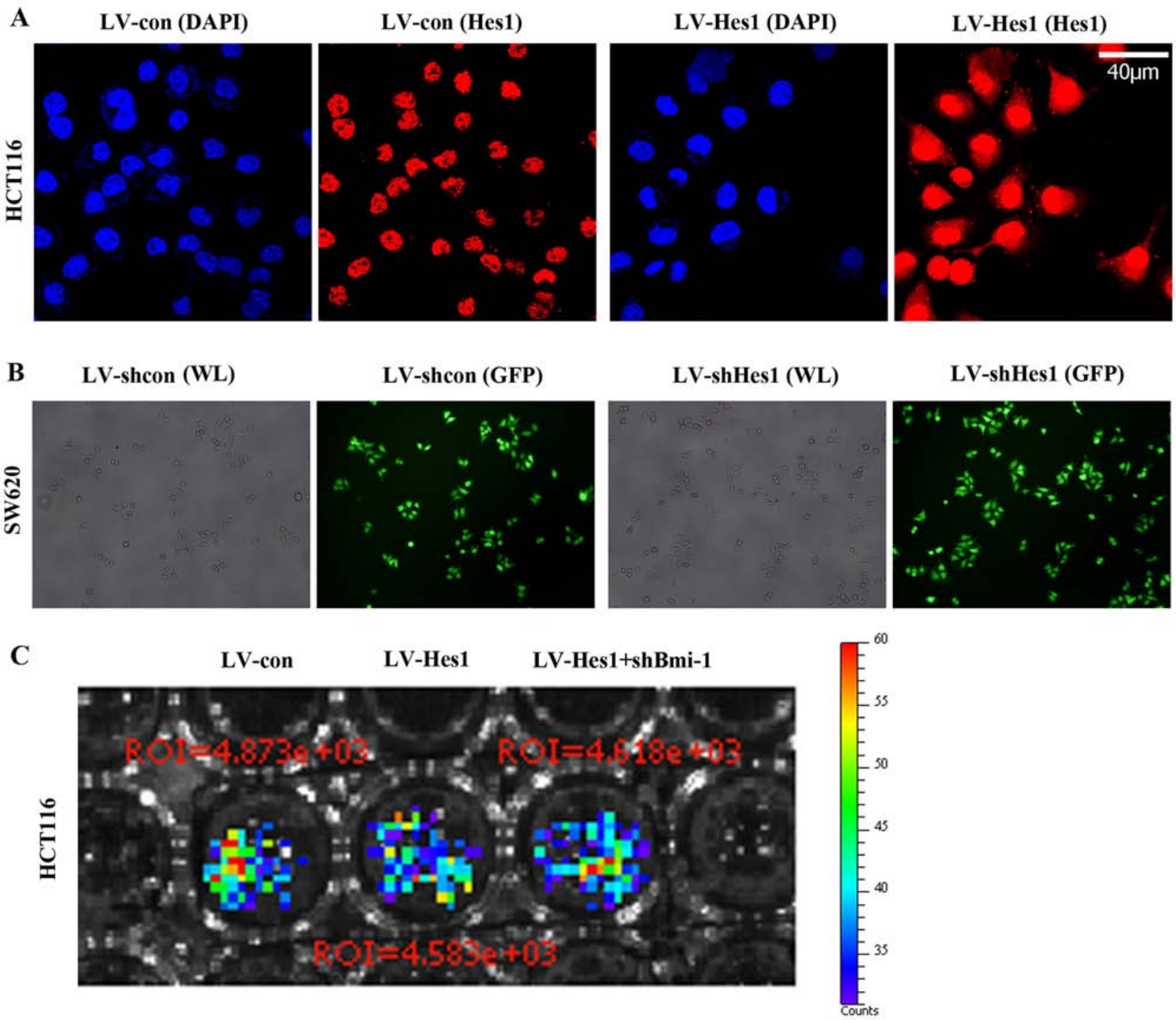

Supplementary Figure S5: Stable cell line establishment. A. Expression of Hes1 in Hes1-overexpressing HCT116 cells detected by Immunofluorescence. **B.** GFP of shcon and shHes1 plasmid vectors in SW620 cells. Original magnification, × 100. **P* < 0.05, #*P* < 0.01 compared with control. **C.** Signal of luciferase in Hes1-overexpressing HCT116 cells and Bmi-1-silencing Hes1-overexpressing HCT116 cells, as well as GFP of "LV-Hes1+shBmi-1" cells. WL: white light; GFP: The green fluorescent protein.

Supplementary Table S1: Primers used in qPCR

Gene	Forward primer (5′-3′)	Reverse primer (5′-3′)
Hes1	ACGTGCGAGGGCGTTAATAC	GGGGTAGGTCATGGCATTGA
Bmi-1	CGTGTATTGTTCGTTACCTGGA	TTCAGTAGTGGTCTGGTCTTGT
PTEN	TGGATTCGACTTAGACTTGACCT	GGTGGGTTATGGTCTTCAAAAGG
E-cadherin	TGCCCAGAAAATGAAAAAGG	GTGTATGTGGCAATGCGTTC
N-cadherin	ACAGTGGCCACCTACAAAGG	CCGAGATGGGGTTGATAATG
Vimentin	GACGCCATCAACACCGAGTT	CTTTGTCGTTGGTTAGCTGGT
Snail	TCGGAAGCCTAACTACAGCGA	AGATGAGCATTGGCAGCGAG
Twist	GTCCGCAGTCTTACGAGGAG	GCTTGAGGGTCTGAATCTTGCT
